# (Bio)active Compounds in Daisy Flower (*Bellis perennis*)

**DOI:** 10.3390/molecules28237716

**Published:** 2023-11-22

**Authors:** Anna-Lena Albien, Timo D. Stark

**Affiliations:** Chair of Food Chemistry and Molecular Sensory Science, Technical University of Munich, Lise-Meitner-Str. 34, 85354 Freising, Germany; ge86bey@mytum.de

**Keywords:** belli/perennisaponins, anticancerogenic, antimicrobial, antidepressive, anxiolytic

## Abstract

The common daisy (*Bellis perennis*) belongs to the family Asteraceae and, in recent years, some new research has been published on the bioactive compounds and biological activities of its extracts. In 2014, the knowledge was partially summarized, but several new studies have been published in the last nine years. In addition, the substances were tabularly consolidated to give a comprehensive overview of over 310 individual components, compound classes, and bioactivities, as well as their accurate plant organ origin. The latest results have shown that the plant has antioxidative, antimicrobial, anticancerogenic, wound healing, antidepressive, anxiolytic, nephroprotective, and insulin mimetic effects, as well as an effect on lipid metabolism. Some studies in the field of homeopathy were also listed. Ideally, a biological effect and one or several compound(s) can be correlated. However, the compounds of the extracts used have often been qualified and quantified, but it remains unclear which of these substances have an activity. The works often stick at the level of the crude extract or a fraction, but not at a single purified and tested compound and, consequently, they are hampered by a missing comprehensive bioactivity workflow. This review provides a critical overview and gaps and offers a basis for further research in this area.

## 1. Introduction

**Figure 1:**
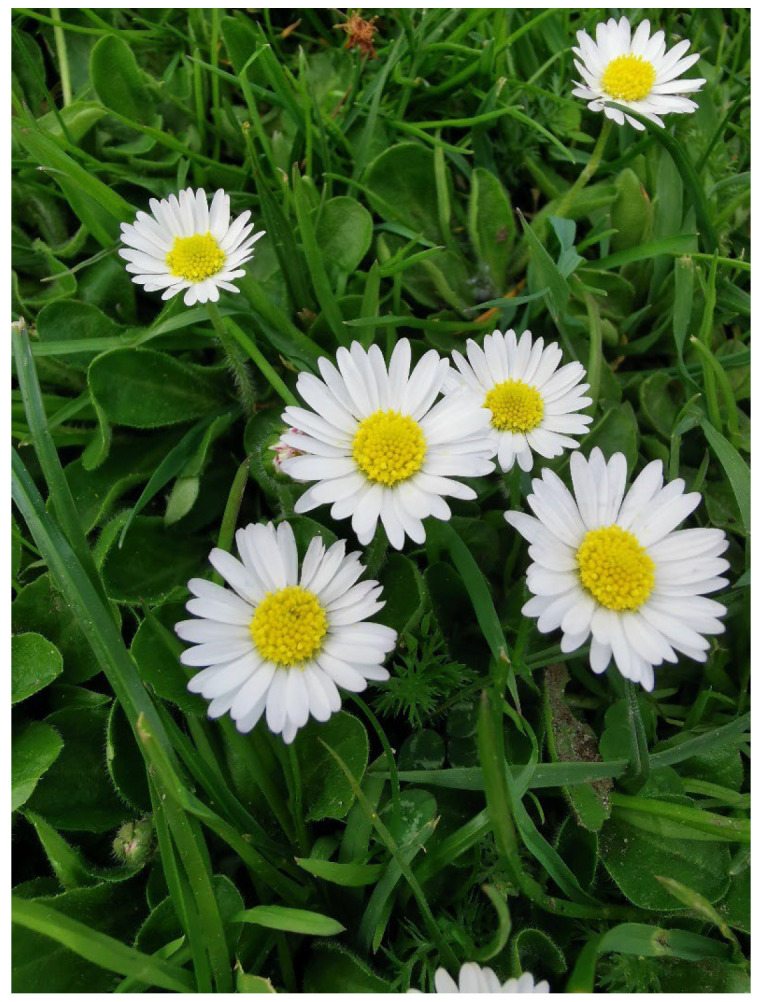
*B. perennis*.

The common daisy (Figure 1), whose botanical name is *Bellis perennis*, is located in Europe, Northern America, and Central Asia. *B. perennis* belongs to the family Asteraceae. The pseudanthium is very characteristic of this family. The disc florets are yellow in color, while the ray florets are usually white to pink in color but can also have a deep red color. The maximum height is 25 cm, the leaves are round shaped and arranged in a basal rosette, and the stem does not have any leaves [1].

The Asteraceae family includes more than a thousand species, including some well-known medicinal plants like milk thistle (*Silybum marianum*), chamomile (*Matricaria chamomilla*), and marigold (*Calendula officinalis*) [1]. *B. perennis* has also been used as a medicinal plant in traditional medicine and, over the past 25 years, antioxidative, anxiolytic, antidepressive, antihyperlipidemic, anticancerogenic, and antimicrobial effects are described in the literature [2]. The majority of studies have been conducted with the aqueous, methanolic, or ethanolic extract of the plant. But, a correlation between active ingredients and biological activity has not always been directly established. However, many secondary plant constituents have also been identified and described in the literature [3,4]. In recent years, research has been conducted on individual constituents of *B. perennis* on the basis of their biological activity, thus opening up further possible therapies for diseases, such as cancer or hyperlipidemia [5].

The goal of this work is to summarize and list the findings of the biological activity of the extracts with the known ingredients. In addition, the most recent results are presented.

## 2. (Bio)activity

### 2.1. Antioxidative Activity

In 2009, Kavalcıoğlu et al. compared the aqueous and methanolic extract of *B. perennis* in terms of antioxidative capacity. They used the 1,1-diphenyl-2-picryl-hydrazyl (DPPH) radical assay with linoleic acid as a blank and butylated hydroxytoluene as a positive control. Both extracts showed reduced activity, while the aqueous extracts had a higher DPPH scavenging activity with 85.8% at 102.5 µg/mL. They also described the reductive capabilities (as measured against the absorbance at 700 nm) of the extracts compared to ascorbic acid. The absorbances at 700 nm at a concentration of 77 μg/mL of the methanol and aqueous extracts of *B. perennis* were specified as 0.118 ± 0.022 and 0.174 ± 0.010 compared to the control with 0.016 ± 0.003 and ascorbic acid with 3.625 ± 0.003 [6].

In 2010, Siatka and Kašparová investigated seasonal variation in the *B. perennis* flavonoid and phenolic content and subsequent antioxidant activity changes by means of the DPPH radical test. The variation in content and radical scavenging activity was small. They described a correlation between the total phenolics and the antioxidant activity, but there was no correlation between the total flavonoids and the antioxidant activity [7].

Marques et al. published the results of the antioxidant potential of the *B. perennis* ethanolic flower extract. They tested the removal capacity against hydroxy radicals and nitric oxide, as well as the prevention of the formation of thiobarbituric acid reactive substances (TBARS). In principle, the antioxidant potential increased with higher concentrations of the extracts [8].

Further, isolated fractions of flowers from *B. perennis* were evaluated, in which the flavonoid apigenin-7-*O*-glucopyranoside showed antioxidant activity in all methods (Table 1) [9].

**Figure 2:**
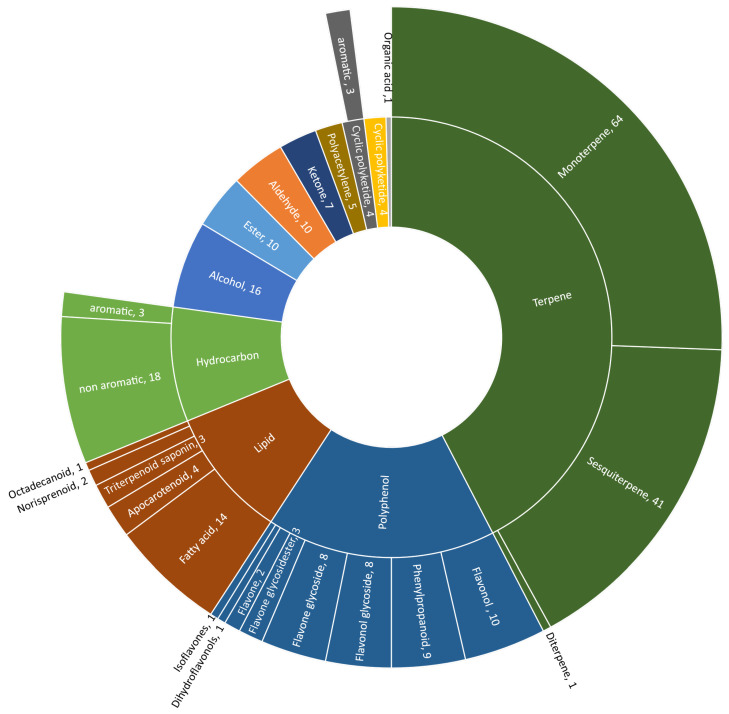
Sunburst plot of substance and sub-substance classes of (bio)active metabolites identified in *B. perennis*.

Karakas et al. extracted the aerial parts of *B. perennis* with different methods using Soxhlet extraction with hexane, dichloromethane, methanol and water, water bad extraction with hot and cold water, ethanol, methanol, and acetone, as well as decoction, infusion, and liquid–liquid extraction with *n*-butanol and ethyl acetate. For the antioxidative activity the radical scavenging activity with DPPH, total phenolic content via a Folin–Ciocalteu reagent, oxygen radical absorbing capacity (ORAC) for in vitro, and 20,70-dichlorofluorescin–diacetate (DCFH-DA) cell-based assays for ex vivo testing were used. The methanol extract of leaves and flowers of wild-grown plants showed higher DPPH radical scavenging activity than in vitro-derived leaves. The total phenolic content was the highest in the methanolic extract of the leaves with 10.60 g/100 g, but the ethyl acetate and *n*-butanol extracts were not determined. The ethyl acetate fraction of the flowers showed the highest ORAC value of 9.05 µmol Trolox/mg. Further, antioxidative activity was tested by DCFH-DA ex vivo, and the *n*-butanol fraction revealed a half-maximal Inhibitory Concentration (IC_50_) of 1.00 µg/mL compared to the control (Trolox, IC_50_ 0.15 µg/mL). The phenolic compounds were determined by LC-ESI-MS/MS. Gallic acid, vanillic, caffeic and *p*-coumaric acid, taxifolin, coumarin, luteolin-7-*O*-β-d-glucoside, rutin, myricetin, kaempferol 3-*O*-β-d-glucopyranoside, quercetin, genistein, and apigenin were found in the methanolic and dichloromethane flower extracts and fraction (Table 1, Figure 2). The highest number of phenolic compounds was found in the ethyl acetate extract by LC-ESI-MS/MS [10].

The anti-inflammatory and anti-arthritic potential of *B. perennis* along with *Asparagus officinalis*, *Daucus carota,* and *Sambucus nigra* were investigated by Marelli et al. in 2020. The methanolic extract of aerial parts of the plants was evaluated for in vitro antioxidant activity by DPPH, 2,20-Azino-bis-3-ethylbenzthiazoline-6-sulphonic acid (ABTS), ferric reducing antioxidant power ferrozine (FRAP-FZ), and the β-carotene bleaching test, as well as their ability to inhibit NO production in vitro. DPPH and ABTS assays showed IC_50_ values of 168.4 and 74.69 µg/mL for *B. perennis*, with only *S. nigra* highlighting stronger radical scavenging activity. Compared to *S. nigra* and *A. officinalis,* the IC_50_ value of 557.89 µg/mL for FRAP-FZ and 78.45 µg/mL in the β-carotene bleaching test indicated a less effective antioxidant property for the *B. perennis* extract. Also, it inhibited nitric oxide production in lipopolysaccharide-stimulated murine macrophage RAW 264.7 cells with an IC_50_ value of 193.1 µg/mL. Marelli et al. used GC-MS (*n*-hexane fraction) and HPTLC (polar residue of the methanolic extracts after fractionation with *n*-hexane) to identify and quantify the compounds in the extracts and observed that the total phenolic and flavonoid content in the raw extract was related to antioxidant and radical scavenging activity. The main compounds in the *n*-hexane fraction of *B. perennis* were alpha-linolenic and linoleic acid. Palmitic, myristic and stearic acid, neophtadiene, stigmasta-7,22-dien-3-ol, and 2-phytene were also identified (Table 1) [11].

Very recently, Karić et al. investigated the antioxidant capacity of commercially available daisy extract (no further details mentioned) using DPPH and FRAP assays. They found out that the FRAP value of 742.11 µmol/g of the sample indicated a good reducing ability in relation to the control ascorbic acid, and the IC_50_ of 0.097 mg/mL in the DPPH assay showed a high capacity to neutralize radicals [12].

### 2.2. Antimicrobial Effects

In 1989, the ethanolic extract of *B. perennis* was tested for antifungal agents by Deseveday et al. [13]. The extract inhibited the growth of *Ceratocystis ulmi* in vitro and also stopped the fungal infection of elms compared to diseased control elms. Activity-guided fractionation yielded polygalacin D as the most effective antifungal compound [13].

Willigmann et al. extracted *B. perennis* with 80% methanol and purified the extract to the crude saponins and saponin esters. It showed in vitro antimycotic activity against *Candida albicans*, *Trichophyton rubrum,* and *T. mentagrophytes* by means of the agar diffusion tests. Saponin esters also inhibited *Aspergillus niger*. An inhibition of *Trichophyton tonsurans*, *T. terestra*, *T. mentagrophytes*, *T. rubrum*, *Microsporum canis*, *M. gypseum*, *Candida krusei,* and *C. albicans* was found in slant agar test tubes, whereas bellissaponins 1 and 2 were identified in the crude saponin fraction via HPLC and TLC [14].

Polyacetylenes have been evaluated for antimicrobial activity in vitro by Avato et al. [15]. Deca-4,6-dynoic acid was the most active component against Gram-positive bacteria, with a mean minimal inhibition concentration (MIC) of 0.35 mg/mL. An MIC of 0.125 mg/mL was found against *Staphylococcus aureus* ATCC 25923 and *Enterococcus faecalis*. Growth of the yeasts *C. albicans*, *Candida tropicalis,* and *Saccharomyces cerevisea* was also inhibited. A synthetic derivative, deca-4,6-diyne-1,1-*O*-dioic acid, indicated antimicrobial activity against Gram-negative bacteria [15].

Karakas et al. tested 19 extracts that are mentioned above against Gram-positive (*S. aureus*, *Streptococcus pyogenes*, *Staphylococcus epidermidis*) and Gram-negative (*Serratia marcescens*, *Salmonella typhimurum*, *Pseudomonas aeruginosa*, *Proteus vulgaris*, *Klebsiella pneumonia*, *Enterobacter cloacae*, *Escherichia. coli*) bacteria using the disc diffusion assay. While all Gram-positive bacteria were inhibited, only *E. cloacae* was inhibited by *B. perennis*. The ethyl acetate fraction was most effective against *S. pyogenes* (12.4 mm inhibition area) and *E. cloacea* (15.9 mm), and the methanolic fraction was most effective against *S. aureus* (16.0 mm) and *S. epidermis* (23.2 mm). The inhibition zone of the most effective positive control was two to three times larger [10].

Also, Kavalcıoğlu et al. and Karić et al. tested *B. perennis* for antimicrobial activity. Kavalcıoğlu et al. checked volatiles and methanolic extracts against *E. coli*, *S. aureus*, *P. aeruginosa*, *Enterobacter aerogenes*, *P. vulgaris*, *S. typhimurium*, and *C. albicans*. Karić et al. investigated commercially available *B. perennis* extracts against the bacteria *E. coli*, *S. aureus*, *P. aeruginosa,* and *E. faecalis* using the disc diffusion assay. In both studies, no inhibition of the bacteria and *C. albicans* was observed [6,12].

### 2.3. Effects on Enzymes

Marques et al. examined apigenin-7-*O*-glucopyranoside for inhibiting acetylcholinesterase (AChE) activity. Rivastigmine was used as a standard and the buffer as a negative control, and the isolated substance revealed a concentration of 0.1% and an inhibition of 76.86%, representing an IC_50_ of 1.91 µmol/L [9].

Further, the ethanolic extract from *B. perennis* flowers and an isolated fraction, namely isorhamnetin 3-*O*-β-d-(6′’-acetyl)-galactopyranoside, was tested in vivo in mice and in vitro for the inhibition of AChE using the methods of Ellmann et al. [16]. Both the ethanolic extract and isorhamnetin 3-*O*-β-d-(6′’-acetyl)-galactopyranoside indicated AChE inhibition in vivo for mice. The ethanolic extract was used in a concentration of 50, 100, and 150 mg/kg, as well as the isolated polyphenol in 10 mg/kg, respectively. Compared to the positive control rivastigmine (400 mg/kg, 5.69 nM/mg) and negative control (10.03 nM/mg), a reduction in AChE activity was measured (1.91, 1.66, 1.79 nM/mg and 0.89 nM/mg). Additionally, the isolated fraction inhibited the AChE in vitro with an IC_50_ of 1.49 mM [17].

Karić et al. tested commercial *B. perennis* extract for the in vitro inhibition of the tyrosinase enzyme. By monitoring the formation of dopachrome at a wavelength of 492 nm, the inhibition of the enzyme, with a calculated IC_50_ of 179.19 mM, was determined [12].

### 2.4. Anticancerogenic Effects

Li et al. isolated bellisosides A–F (Table 1) from methanolic *B. perennis* root extract and tested the new isolates and the known bellissaponin BS2 for cytotoxic activity against HL-60 human promyelocytic leukemia cells. The cells were treated with each substance for 72 h and the cell growth was measured using the 3-(4,5-dimethylthiazol-2-yl)-2,5-diphenyl-tetrazolium bromide (MTT) assay. Compared to cisplatin (IC_50_: 1.8 µM) as a positive control, all substances reduced cell growth, and strong cytotoxicity of bellisoside E and F with IC_50_ values of 1.4 µM and 0.5 µM was observed. Only these two saponins contained a long-chain acyl group in the chemical structure; consequently, the authors presumed that this molecular feature is related to elevated cytotoxic activity [18].

In 2014, Karakas et al. published their discovery of an oleanane-type saponin as an antitumor drug. The butanol extract of *B. perennis* flowers and stems was separated into nine fractions by liquid chromatography. These fractions were tested for antitumor activity using the potato disc method modified by McLaughlin [19,20,21]. Fraction “G” as well as “G3” highlighted the highest tumor inhibition compared to camptothecin as a positive control. A perennisaponin A–M-like compound was found in the purified fraction G3, which was characterized by means of spectroscopic (1/2D-NMR) and spectrometric (LC-ESI-TOF-MS) methods.^5^ Unfortunately this structure was not specified by IUPAC nomenclature or via trivial name; consequently, it is not listed in Table 1.

One year later, Karakas et al. compared different extracts (hexane, dichloromethane, methanol, water) from different plants (*B. perennis, Convolvulus galaticus, Trifolium Pannonicum subsp. elongatum, Lysimachia vulgaris*) for antitumor activity. The antiproliferative activity against breast cancer (MCF-7) and human hepatocellar carcinoma (HepG2/C3A) cell lines were investigated by means of the MTT assay. The methanolic extract of aerial parts from *B. perennis* indicated the highest activity of all plants tested and resulted in an IC_50_ of 71.6 on MCF-7 cell lines and 73.9 µg/mL for HepG2/C3A, respectively. The phenolic compounds from the methanolic extract were analyzed via HPLC and the following compounds; namely, gallic acid, caffeic acid, rutin, kaempferol, myricetin, quercetin, and apigenin were identified (Table 1) [22].

Ninomiya et al. examined the methanolic *B. perennis* flower extract as well as perennisaponins A-T for antiproliferative activity against human gastric cancer cell lines (HSC-2, HSC-4, MKN-45) using the MTT assay. All components highlighted antiproliferative activity, whereas perennisaponin O was outstanding, with IC_50_ values of 11.2 against HSC-2, 14.3 against HSC-4 and 6.9 µM against MKN-45. Annexin-V/7-aminoactinomycin D (7-ADD) staining as a marker of early and late apoptotic events was used to determine the apoptosis-inducing effects of perennisaponin O on HSC-2 cells and indicated concentration dependencies between 3 and 30 µM [23].

### 2.5. Effects on Skin and Wound Healing

Karakas et al. investigated the wound-healing properties of *B. perennis* by topical application on rats. In this approach, flowers and pedicels of the plant were extracted with ethanol and fractionated with *n*-butanol. Hydrophilic ointment was used as a base formulation and also as a control. Six 4 mm wounds were inflicted on the rats by a punch biopsy. Two wounds were treated once a day with the hydrophilic ointment, two with none at all, and two with the hydrophilic ointment containing the *B. perennis* butanolic fraction. Complete wound closure (100%) was observed after 30 days of treatment with hydrophilic ointment containing the fraction, whereas the wounds treated only with hydrophilic ointment were close to 85% and the non-treated wounds to 87%. Histopathological differences were also observed favoring *B. perennis* treatment [24].

Morikawa et al. found out that the methanolic *B. perennis* flower extract promoted collagen synthesis activity in normal human dermal fibroblasts (NHDFs). The methanolic extract (10 µg/mL) indicated a higher collagen content (147.3%) than the control (100%). Perennisoside I–III, VII, IX, and XI–XIX, bernadioside B2, bellidioside A, bellissaponin BS6, BS1, and perennisaponin B, F, and K, as well as bellisoside D, E, and F could be isolated from the active extract (Table 1, Figure 2). These pure substances were also tested in cell culture for collagen synthesis-promoting activity and cell toxicity via MTT. Perennisosides XVII, I, II, VII, IX, and XI, as well as asterbatanoside D, bernardioside B2, and bellissaponins BS5 and BS9 revealed higher activity in used concentrations of 10–30 µM without showing cytotoxicity than asiaticoside (138.1% at 100 µM) and madecassoside (113.5% at 100 µM), which are known for their collagen-promoting activity [25].

In 2021, Souza de Carvalho et al. revealed an in vitro photoprotective and immunomodulatory effect of commercial *B. perennis* extract (Biofarma, Brazil). A human skin keratinocyte (HaCaT) cell culture was incubated with 0.01, 0.1, or 1% *B. perennis* extract and submitted to UV radiation at 365 nm for 60 min. After 24 h, the HaCaT was investigated for cell viability by means of the MTT assay and lactate dehydrogenase (LDH) activity, cleaved caspase-3, cyclooxygenase-2, and reactive oxygen species (ROS). The dosage of interleukin-6 (IL-6) was detected by an enzyme-linked immunosorbent assay (ELISA), and the cells were also analyzed for catalase, glutathione peroxidase, and superoxide dismutase activity. Compared to untreated cells as a control, *B. perennis* extract indicated an increase in cell viability, a lower level of liberated caspase, ROS, and IL-6, and higher activity of catalase and glutathione peroxidase. A difference in COX-2 expression was not observed. As the *Polypodium leucotomos* extract (Biofarma) is known for photoprotective effects in vitro and in vivo, it was used as a positive control. The 1% *P. leucotomos* and 1% *B. perennis* extracts showed similar effects on the cells [26].

### 2.6. Antidepressive/Anxiolytic Effects

To evaluate the potential antidepressive effect of *B. perennis* flowers and pedicels, adult and juvenile Wistar albino rats were treated with both aqueous extracts [27]. Therefore, the extracts were injected daily at the same time in a dosage of 20 or 60 mg/kg and a control group received saline, respectively. The tests for anxiety-like behavior used the open field test as well as the elevated plus maze test, and spatial memory was investigated by the Morris water maze test two hours after the dosage. A change in the behavior of the rats given the high dose of *B. perennis* extract was observed. In the open field, the rats traveled less distance, spent more time in the center, visited the edge and the center less frequently, and showed less velocity and mobility than the control and low-dose groups. They spent more time in open arms and less time in closed arms, were less mobile, slower, and rotated less frequently than the control and low-dose groups in the elevated plus maze. The effect of *B. perennis* was higher in juveniles in the open field test and higher in adults in the elevated plus maze test. The results in both tests indicated an anxiolytic and anesthetic effect of high-dose *B. perennis* extract. With regard to the anxiolytic effect, *B. perennis* may inhibit the serotonergic activity via the GABAergic system and may act like “benzodiazepines”, which are widely used in reducing anxiety-like behaviors. The anesthetic perspective may have a relevant effect via its anesthetic properties. They found that rats with high-dose injections increased the distance traveled, the time to find the platform and spent in the correct quadrant, the number of entries to the correct quadrant in adults, and the mobility in general but decreased the mobility in adults in the Morris water maze test. The authors interpreted the results as a decrease in learning performance and an increase in spatial memory [27].

In 2012, Marques et al. investigated the anxiolytic and antidepressant effects of ethanolic *B. perennis* flower extracts in mice. They orally administered doses of 50, 100, and 150 mg/kg and observed no changes in behavior after 14 days of treatment. In the open field test, 1.0 mg/kg diazepam intraperitoneally (i.p.) was used as positive control and tween in saline 0.9% (i.p.) as a negative control. Low numbers of crossing, grooming, and rearing indicated an anxiolytic effect in the open field test. Mice, treated with diazepam or *B. perennis*, revealed decreased number of crossing and rearing. Only diazepam injections reduced the number of groomings; the ethanolic extract indicated no changes compared to the negative control. In addition, the mice received an injection of flumazenil, a benzodiazepine antagonist, 15 min before the injection of diazepam or ethanolic extract. Diazepam did not have any effect on the parameters in the open field test compared to the negative control because of the antagonistic effect. The effects of the ethanolic extract were not influenced by flumazenil; therefore, the authors suggested that *B. perennis* had no effect on the GABAergic system. The forced swimming test indicated an antidepressant effect of the tested treatments. Compared to the negative control (tween in saline), the ethanolic extracts at 50, 100, and 150 mg/kg reduced the immobility time by 58, 56, and 68%, respectively. Further, Marques et al. tested selected antidepressants alone or in combination with 150 mg/kg *B. perennis* ethanolic extracts. Imipramine 50 mg/kg (i.p.), paroxetine 20 mg/kg (i.p.), and 0.25 mg/kg reserpine (i.p.) were injected 15 min before the ethanolic extract was administered. The combination of imipramine and the extract showed a greater reduction in immobility time than imipramine or the ethanolic extract alone. The combination of paroxetine and ethanolic extract highlighted no change in immobility time compared with mice treated with the corresponding substance alone. Mice treated with reserpine indicated neither a reduction in immobility time nor a combination of reserpine and ethanolic extract. Reserpine blocked the effect of the ethanolic extract of *B. perennis*. Based on these results, the authors suggested an effect on the noradrenergic activity of the central nervous system [8].

### 2.7. Effects on Lipid Metabolism

A methanolic flower extract of *B. perennis* was tested for the inhibition of serum triglyceride (TG) elevation in olive oil-treated mice by Morikawa et al. The extract (500 mg/kg) and olive oil (5 mL/kg) were orally administered 30 min later. Mice treated with the extract showed reduced serum TG two hours after administration. The same results were obtained by the chromatographically enriched saponin fraction (200 mg/kg) in which seven new triterpene saponins, perennisosides I–VII, and four known saponins, bellidioside A, asterbatanoside D, bernardioside B2, and bellissaponin BS6, were identified (Table 1). Two substances, perennisoside I and II (25, 50, 100 mg/kg), were then individually tested and compared to the hypolipidemic drug clofibrate (125, 250, 500 mg/kg) and the standard lipase inhibitor orlistat (6.25, 12.5, 25 mg/kg). Blood was taken two, four, and six hours after olive oil treatment, and serum TG was enzymatically determined by the commercial triglyceride E test. Perennisoside II showed a greater suppression of serum TG levels than clofibrate at two, four, and six hours after administration, whereas perennisoside I only decreased serum TG at two hours. Mice treated with orlistat had the lowest serum TG levels [28].

Further, Morikawa et al. investigated the in vitro inhibitory effect of the methanolic flower extract and perennisaponins G, H, I, J, K, L, and M on pancreatic lipase activity. Pancreatic lipase activity was assayed as free fatty acid concentration after 30 min incubation with triolein, phosphatidylcholine, sodium taurocholate, and porcine pancreatic lipase. The methanolic extract (IC_50_: 455 µg/mL) and all perennisaponins G-M (IC_50_: 163, 137, 147, 148, 223, 81.4, 195 µM) revealed an inhibitory effect. The authors compared the IC_50_ with theasaponin E1 (270 µM) and orlistat (56 µM). Theasaponin E1 from *Camellia sinensis* is a known inhibitor of pancreatic lipase. The activities of the perennisaponins are stronger than theasaponin E1 but weaker than orlistat [29].

The anti-obesity potential of *B. perennis* along with *Asparagus officinalis*, *Daucus carota,* and *S. nigra* was investigated by Marelli et al. in 2020. The methanolic extracts of aerial parts of the plants were tested for the inhibitory potential of pancreatic lipase by the spectrophotometric method based on the hydrolysis of 4-nitrophenylcaprylate to *p*-nitrophenol measured at 412 nm. Orlistat (20 µg/mL) was used as a positive control. All samples revealed efficacy at 5 mg/mL compared to the control. The best IC_50_ of 1.63 mg/mL was observed for *D. carota* (orlistat: 0.018 mg/mL); the enzyme was inhibited to 93.66%, whereas an inhibition lower than 50% was observed for *B. perennis* and the other plants [11].

### 2.8. Effects on Blood Glucose Levels

Haselgrübler et al. established an in ovo method for testing insulin–mimetic compounds and investigated freshly prepared ethanolic plant extract from *B. perennis,* as well as extracts from an extract library (plant extract collection Kiel in Schleswig-Holstein, PECKISH), a mixture of flowers and leaves (4404), and flowers alone (4407) [30]. The first in vitro screening of CHO-K1 cells expressing the human insulin receptor, and a GLUT4-myc-GFP fusion protein incubated with the extract resulted in an increase in GFP signals of about 8 and 5% of 4404 and 4007, respectively. Compared to insulin as a positive control (≈26%), the freshly prepared extract strongly increased the GFP signal (≈35%). Based on the promising results, Haselgrübler et al. performed the HET-CAM (Hens Egg Test-Chorioallantoic Membrane) assay. The fertilized eggs of unhatched avian embryos were infused with an HBSS buffer or H_2_O containing a 300 mg/L extract and incubated for one and two hours. Blood glucose levels were measured using a blood glucose meter, and Novorapid (3.3 U/mL) was used as a positive control. All three extracts decreased blood glucose levels by approximately 20% after one and 33% after two hours compared to the positive control (≈16% and ≈33%, respectively) in the HBSS buffer. Blood glucose levels were also reduced by extracts dissolved in water by approximately 12% after two hours (Novorapid: ≈25%). The authors qualified and quantified the polyphenolic compounds of the extracts by HPLC-MS and found rutin, hyperoside, isoquercitrin, guaijaverin, avicularin, quercitrin, quercetin, apigenin-7-glucoside, apigenin-7-glucuronide, apigenin, neochlorogenic acid, chlorogenic acid, caffeic acid, kaempferol, and luteolin (Table 1, Figure 2). Kaempferol and luteolin were not quantified due to overlapping retention times. Similar compounds were found in all three extracts, but the freshly prepared extract contained approximately ten times more polyphenolic compounds. The authors concluded that the extracts prepared are rich in polyphenolic compounds, induce GLUT4 translocation in vitro at low concentrations, and effectively reduce blood glucose in living animals [31].

### 2.9. Nephro- and Hematoprotective Effects

In 2018, Zangeneh et al. revealed that aqueous *B. perennis* leaf extract had a protective effect on carbon tetrachloride (CCl_4_)-induced nephrotoxicity in mice. Five groups of mice were prepared. Control group I received 1 mL/kg olive oil i.p. and 0.5 mL of distilled water p.o., and a 1:1 mixture of CCl_4_ and olive oil i.p. were administered to the remaining groups. In addition, 0.5 mL of distilled water was administered in untreated group II and 50, 100, and 200 mg/kg aqueous *B. perennis* extract was administered in groups III, IV, and V p.o. for 45 days. After the treatment, blood samples and the left kidney were taken and examined for structural, hematological, and biochemical changes. In summary, the untreated mice (group II) had a lower body weight as well as lower levels of superoxide dismutase (SOD), catalase (CAT), red blood cells, packed cell volume, mean corpuscular volume, hemoglobin (Hb), mean corpuscular Hb, mean corpuscular Hb concentration, monocyte, and higher levels of creatin, urea and white blood cells, and platelets. The untreated mice also showed some renal hypertrophy. The mice given the *B. perennis* extract revealed enhancement in hematological and biochemical results compared to the control group, which was related to the concentration of the extract. In summary, aqueous *B. perennis* extract indicated hemato and nephroprotective effects in vivo [32].

## 3. Homeopathy

### 3.1. Postpartum Bleeding

Oberbaum et al. published results in a double-blind, placebo-controlled study in which *Arnica montana* and *B. perennis* C6 or C30 were examined for positive effects on mild postpartum bleeding in women. Hb was measured 48 and 72 h postpartum, and the mean Hb levels in the treatment group were 12.7 before birth (placebo: 12.7) and 12.4 72 h postpartum (placebo: 11.6). The authors suggested that homeopathic dosages of *A. montana* and *B. perennis* may reduce postpartum blood loss [33].

### 3.2. Neuroprotective Effect

Khan et al. examined the neuroprotective effects of *B. perennis* and *Hypericum perforatum,* C6 and C30, respectively, in different concentrations (2, 4, 6 µL/mL) on rat pheochromocytoma PC12 cells. Ninety percent ethanol was used as a positive control. MTT and neutral red uptake (NRU) assays were performed after 96 h of treatment. Compared to the positive control (increased cell viability), the cells treated with the homeopathic application had higher levels of glutathione and higher activity of gluthatione peroxidase and reductase, AChE, and monoamine oxidase. The authors concluded that homeopathic levels *B. perennis* and *H. perforatum* had a protective role on PC 12 cells [34].

### 3.3. Seroma Reduction

Lotan et al. performed a randomized, double-blind, and placebo-controlled trial of the homeopathic treatment using *A. montana* and *B. perennis* C30 on reduced seroma following mastectomy and immediate breast reconstruction from surgery to drain removal. The time to drain removal was measured and was lower in the study group (11.1 ± 6.1 days) compared to the placebo (13.5 ± 6.4 days). Homeopathic therapy was proposed as a possible adjunct in the post-operation time for a reduction in drain removal [35].

**Table 1:** Compound name, substance class, organ distribution, extract, and the corresponding literature. Table 1 is available in .xlsx format as Table S1 in Supplementary Materials.

Name	Substance Class	Organ	Extract	Ref.
Hexanol	Alcohol	leaves, flowers	essential oil	[3]
Heptanol	Alcohol	leaves, flowers	essential oil	[3]
Octanol	Alcohol	leaves, flowers	essential oil	[3]
2-Ethyl-l-Hexenol	Alcohol	leaves, flowers	essential oil	[3]
*trans*-2-Hexenol	Alcohol	leaves, flowers	essential oil	[3]
*cis*-3-Hexenol	Alcohol	leaves, flowers	essential oil	[3]
Oct-l-en-3-ol	Alcohol	leaves, flowers	essential oil	[3]
Benzylalcohol	Alcohol	leaves, flowers	essential oil	[3]
2-Phenylethanol	Alcohol	leaves, flowers	essential oil	[3]
Phytol	Alcohol	leaves, flowers, herb	essential oil	[3,6]
1-Hexadecanol	Alcohol	herb, flowers	essential oil	[6]
1-Octadecanol	Alcohol	herb, flowers	essential oil	[6]
1-Octen-3-ol	Alcohol	herb, flowers	essential oil	[6]
1-Dodecanol	Alcohol	herb, flowers	essential oil	[6]
1-Tetradecanol	Alcohol	herb, flowers	essential oil	[6]
Isophytol	Alcohol	herb, flowers	essential oil	[6]
Hexanal	Aldehyde	leaves, flowers, herb	essential oil	[3,6]
Heptanal	Aldehyde	leaves, flowers	essential oil	[3]
Nonanal	Aldehyde	leaves, flowers, herb	essential oil	[3,6]
Decanal	Aldehyde	leaves, flowers, herb	essential oil	[3,6]
*trans*-2-Hexenal	Aldehyde	leaves, flowers	essential oil	[3]
2,4-Hexadienal	Aldehyde	leaves, flowers	essential oil	[3]
Heptadienal	Aldehyde	leaves, flowers	essential oil	[3]
Decadienal	Aldehyde	leaves, flowers	essential oil	[3]
Benzaldehyde	Aldehyde	leaves, flowers	essential oil	[3]
Phenylacet-aldehyde	Aldehyde	leaves, flowers, herb	essential oil	[3,6]
Tetradecanal	Aldehyde	herb, flowers	essential oil	[6]
Pentadecanal	Aldehyde	herb, flowers	essential oil	[6]
(*Z*)-3-Hexenal	Aldehyde	herb, flowers	essential oil	[6]
(*E,Z*)-2,4-Heptadienal	Aldehyde	herb, flowers	essential oil	[6]
(*E,E*)-2,4-Heptadienal	Aldehyde	herb, flowers	essential oil	[6]
(*E*)-2-Nonenal	Aldehyde	herb, flowers	essential oil	[6]
(*E,Z*)-Nonadienal	Aldehyde	herb, flowers	essential oil	[6]
(*E,Z*)-2,4-Decadienal	Aldehyde	herb, flowers	essential oil	[6]
*p*-Vinylguaiacol	Aroma compound	leaves, flowers	essential oil	[3]
2,3-Dihydrobenzofurane	Cyclic polyketides	leaves, flowers	essential oil	[3]
2-Pentylfuran	Cyclic polyketides	herb, flowers	essential oil	[6]
3,4-Dimethyl-5-penthylidene-2(5*H*)furanone	Cyclic polyketides	herb, flowers	essential oil	[6]
3,4-Dimethyl-5-pentyl-5*H*-furan-2-one	Cyclic polyketides	herb, flowers	essential oil	[6]
*cis*-3-Hexenylacetate	Ester	leaves, flowers	essential oil	[3]
*cis*-3-Hexenyl-2-methylbutanoate	Ester	leaves, flowers	essential oil	[3]
Octen-l-ol	Ester	leaves, flowers	essential oil	[3]
Methyl palmitate	Ester	leaves, flowers	essential oil	[3]
Isopropyl palmitate	Ester	leaves, flowers	essential oil	[3]
Methyl linoleate	Ester	leaves, flowers, herb	essential oil	[3,6]
Methyl linoleate	Ester	leaves, flowers	essential oil	[3]
9,12-Hexadecadienoic acid methylester	Ester	herb, flowers	essential oil	[6]
Ethyl linoleate	Ester	herb, flowers	essential oil	[6]
Methyl ethyl hexadeconoate	Ester	herb, flowers	essential oil	[6]
1,2,3-Trimethylbenzene	Hydrocarbon (aromatic)	leaves, flowers	essential oil	[3]
Naphthalene	Hydrocarbon (aromatic)	leaves, flowers, herb	essential oil	[3,6]
Naphthalene-l,2-dihydro-l,l,6-trimethyl	Hydrocarbon (aromatic)	leaves, flowers	essential oil	[3]
*trans*-Decahydronaphthalene	Hydrocarbon (non-aromatic)	leaves, flowers	essential oil	[3]
*cis*-Cyclododecene	Hydrocarbon (non-aromatic)	leaves, flowers	essential oil	[3]
Undecane	Hydrocarbon (non-aromatic)	leaves, flowers	essential oil	[3]
Dodecane	Hydrocarbon (non-aromatic)	leaves, flowers	essential oil	[3]
Tridecane	Hydrocarbon (non-aromatic)	leaves, flowers	essential oil	[3]
Tetradecane	Hydrocarbon (non-aromatic)	leaves, flowers, herb	essential oil	[3,6]
Hexadecane	Hydrocarbon (non-aromatic)	leaves, flowers, herb	essential oil	[3,6]
Heptadecane	Hydrocarbon (non-aromatic)	leaves, flowers, herb	essential oil	[3,6]
Octadecane	Hydrocarbon (non-aromatic)	leaves, flowers	essential oil	[3]
Nonacosan	Hydrocarbon (non-aromatic)	herb, flowers	essential oil	[6]
Heptacosan	Hydrocarbon (non-aromatic)	herb, flowers	essential oil	[6]
Dimethyl tetradecane	Hydrocarbon (non-aromatic)	herb, flowers	essential oil	[6]
Pentadecane	Hydrocarbon (non-aromatic)	herb, flowers	essential oil	[6]
Nonadecane	Hydrocarbon (non-aromatic)	herb, flowers	essential oil	[6]
Eicosane	Hydrocarbon (non-aromatic)	herb, flowers	essential oil	[6]
Heneicosane	Hydrocarbon (non-aromatic)	herb, flowers	essential oil	[6]
Tricosan	Hydrocarbon (non-aromatic)	herb, flowers	essential oil	[6]
Pentacosane	Hydrocarbon (non-aromatic)	herb, flowers	essential oil	[6]
6-Methyl-5-hepten-2-one	Ketone	leaves, flowers	essential oil	[3]
Oct-3-en-2-one	Ketone	leaves, flowers	essential oil	[3]
Nonan-2-one	Ketone	leaves, flowers	essential oil	[3]
Pentadecan-2-one	Ketone	leaves, flowers, herb	essential oil	[3,6]
Heptadecan-2-one	Ketone	leaves, flowers	essential oil	[3]
Pentadecan-2-one-6,10,14-trimethyl	Ketone	leaves, flowers	essential oil	[3]
Acetophenone	Ketone	leaves, flowers	essential oil	[3]
β-Damascenone	Lipid (Apocarotenoid)	leaves, flowers	essential oil	[3]
β-Ionone	Lipid (Apocarotenoid)	leaves, flowers, herb	essential oil	[3,6]
Dihydroactinidiolide	Lipid (Apocarotenoid)	leaves, flowers	essential oil	[3]
Cyclocitral	Lipid (Apocarotenoid)	herb, flowers	essential oil	[6]
Octanoic acid	Lipid (Fatty acids)	leaves, flowers	essential oil	[3]
Nonanoic acid	Lipid (Fatty acids)	leaves, flowers	essential oil	[3]
Decanoic acid	Lipid (Fatty acids)	leaves, flowers	essential oil	[3]
Undecanoic acid	Lipid (Fatty acids)	leaves, flowers	essential oil	[3]
Lauric acid	Lipid (Fatty acids)	leaves, flowers	essential oil	[3]
Tridecanoic acid	Lipid (Fatty acids)	leaves, flowers	essential oil	[3]
Myristic acid	Lipid (Fatty acids)	leaves, flowers, aerial parts	essential oil, methanolic extract	[3,11]
Pentadecanoic acid	Lipid (Fatty acids)	leaves, flowers	essential oil	[3]
Palmitic acid	Lipid (Fatty acids)	leaves, flowers, aerial parts	essential oil, methanolic extract	[3,11]
Heptadecanoic acid	Lipid (Fatty acids)	leaves, flowers, herb	essential oil	[3,6]
Stearic acid	Lipid (Fatty acids)	leaves, flowers, aerial parts	essential oil, methanolic extract	[3,11]
Palmitoleic acid	Lipid (Fatty acids)	leaves, flowers	essential oil	[3]
Linoleic acid	Lipid (Fatty acids)	leaves, flowers, aerial parts	essential oil, methanolic extract	[3,11]
Linolenic acid	Lipid (Fatty acids)	leaves, flowers, aerial parts	essential oil, methanolic extract	[3,11]
(*E*)-Theaspirane	Lipid (Norisprenoid)	herb, flowers	essential oil	[6]
(*Z*)-Theaspirane	Lipid (Norisprenoid)	herb, flowers	essential oil	[6]
*cis*-Jasmone	Lipid (Octadecanoids)	herb, flowers	essential oil	[6]
Perennisaponin A	Lipid (Triterpene saponin)	flowers	methanolic extract	[23,36]
Perennisaponin B	Lipid (Triterpene saponin)	flowers	methanolic extract	[23,36]
Perennisaponin C	Lipid (Triterpene saponin)	flowers	methanolic extract	[23,36]
Perennisaponin D	Lipid (Triterpene saponin)	flowers	methanolic extract	[23,36]
Perennisaponin E	Lipid (Triterpene saponin)	flowers	methanolic extract	[23,36]
Perennisaponin F	Lipid (Triterpene saponin)	flowers	methanolic extract	[23,36]
Perennisaponin G	Lipid (Triterpene saponin)	flowers	methanolic extract	[23,29]
Perennisaponin H	Lipid (Triterpene saponin)	flowers	methanolic extract	[23,29]
Perennisaponin I	Lipid (Triterpene saponin)	flowers	methanolic extract	[23,29]
Perennisaponin J	Lipid (Triterpene saponin)	flowers	methanolic extract	[23,29]
Perennisaponin K	Lipid (Triterpene saponin)	flowers	methanolic extract	[23,29]
Perennisaponin L	Lipid (Triterpene saponin)	flowers	methanolic extract	[23,29]
Perennisaponin M	Lipid (Triterpene saponin)	flowers	methanolic extract	[23,29]
Perennisaponin N	Lipid (Triterpene saponin)	flowers	methanolic extract	[23]
Perennisaponin O	Lipid (Triterpene saponin)	flowers	methanolic extract	[23]
Perennisaponin P	Lipid (Triterpene saponin)	flowers	methanolic extract	[23]
Perennisaponin Q	Lipid (Triterpene saponin)	flowers	methanolic extract	[23]
Perennisaponin R	Lipid (Triterpene saponin)	flowers	methanolic extract	[23]
Perennisaponin S	Lipid (Triterpene saponin)	flowers	methanolic extract	[23]
Perennisaponin T	Lipid (Triterpene saponin)	flowers	methanolic extract	[23]
Bellissaponin BS1	Lipid (Triterpene saponin)	flowers	methanolic extract	[23,36]
Perennisoside I	Lipid (Triterpene saponin)	flowers	methanolic extract	[25,28,36]
Perennisoside II	Lipid (Triterpene saponin)	flowers	methanolic extract	[25,28,36]
Perennisoside III	Lipid (Triterpene saponin)	flowers	methanolic extract	[25,28,36]
Perennisoside IV	Lipid (Triterpene saponin)	flowers	methanolic extract	[25,28,36]
Perennisoside V	Lipid (Triterpene saponin)	flowers	methanolic extract	[25,28,36]
Perennisoside VI	Lipid (Triterpene saponin)	flowers	methanolic extract	[25,28,36]
Perennisoside VII	Lipid (Triterpene saponin)	flowers	methanolic extract	[25,28,36]
Besysaponin UD2	Lipid (Triterpene saponin)	flowers	methanolic extract	[25,28]
Bellidioside A	Lipid (Triterpene saponin)	flowers	methanolic extract	[25,28,36]
Asterbatanoside D	Lipid (Triterpene saponin)	flowers	methanolic extract	[25,28,36]
Bernardioside B2	Lipid (Triterpene saponin)	flowers	methanolic extract	[25,28,36]
Bellissaponin BS6	Lipid (Triterpene saponin)	flowers	methanolic extract	[25,28,36]
Perennisoside VIII	Lipid (Triterpene saponin)	flowers	methanolic extract	[25]
Perennisoside IX	Lipid (Triterpene saponin)	flowers	methanolic extract	[25]
Perennisoside X	Lipid (Triterpene saponin)	flowers	methanolic extract	[25]
Perennisoside XI	Lipid (Triterpene saponin)	flowers	methanolic extract	[25]
Perennisoside XII	Lipid (Triterpene saponin)	flowers	methanolic extract	[25]
Perennisoside XIII	Lipid (Triterpene saponin)	flowers	methanolic extract	[25]
Perennisoside XIV	Lipid (Triterpene saponin)	flowers	methanolic extract	[25]
Perennisoside XV	Lipid (Triterpene saponin)	flowers	methanolic extract	[25]
Perennisoside XVI	Lipid (Triterpene saponin)	flowers	methanolic extract	[25]
Perennisoside XVII	Lipid (Triterpene saponin)	flowers	methanolic extract	[25]
Perennisoside XVIII	Lipid (Triterpene saponin)	flowers	methanolic extract	[25]
Perennisoside XIX	Lipid (Triterpene saponin)	flowers	methanolic extract	[25]
Bellisoside D	Lipid (Triterpene saponin)	flowers, roots	methanolic extract	[18,25,36]
Bellisoside E	Lipid (Triterpene saponin)	flowers, roots	methanolic extract	[18,25,36]
Bellisoside F	Lipid (Triterpene saponin)	flowers, roots	methanolic extract	[18,25,36]
Bellissaponin BS5	Lipid (Triterpene saponin)	flowers	methanolic extract	[25]
Bellissaponin BS9	Lipid (Triterpene saponin)	flowers	methanolic extract	[25]
Polygalacin D	Lipid (Triterpene saponin)	whole plant	ethanolic extract	[13]
Bellissaponin 1	Lipid (Triterpene saponin)	whole plant	methanolic extract	[14]
Bellissaponin 2	Lipid (Triterpene saponin)	whole plant	methanolic extract	[14]
Bellisoside A	Lipid (Triterpenoid saponin)	roots	methanolic extract	[18]
Bellisoside B	Lipid (Triterpenoid saponin)	roots	methanolic extract	[18]
Bellisoside C	Lipid (Triterpenoid saponin)	roots	methanolic extract	[18]
Hexandicarboxilyc acid	Organic acids	leaves, flowers	essential oil	[3]
Benzoic acid	Organic acids (aromatic carboxylic acid)	leaves, flowers	essential oil	[3]
Phenylacetic acid	Organic acids (aromatic carboxylic acid)	leaves, flowers	essential oil	[3]
3,5-Dimethylphenol	Organic acids (aromatic carboxylic acid)	leaves, flowers	essential oil	[3]
Methyldeca-4,6-diynoate	Polyacetylenes	leaves, flowers, aerial organs	essential oil	[3,15]
Lachnophyllum ester	Polyacetylenes	leaves, flowers	essential oil	[3]
Matricaria ester	Polyacetylenes	leaves, flowers	essential oil	[3]
Deca-4,6-diynoic acid	Polyacetylenes	leaves, flowers, aerial organs	essential oil	[3,15]
Lachophyllum acid	Polyacetylenes	leaves, flowers	essential oil	[3]
Gallic acid monohydrate	Polyphenol	aerial parts	methanolic extract, DCM extract	[10]
Taxifolin hydrate	Polyphenol (Dihydroflavonols)	aerial parts	methanolic extract, DCM extract	[10]
Apigenin-7-glucoside	Polyphenol (Flavone glycosides)	plants	ethanolic extract	[31]
Apigenin-7-glucuronide	Polyphenol (Flavone glycosides)	plants	ethanolic extract	[31]
Apigenin-7-*O*-glucopyranoside	Polyphenol (Flavone glycosides)	flowers	ethanolic extract	[9]
Luteolin-7-*O*-β-d-glucoside	Polyphenol (Flavone glycosides)	aerial parts	DCM extract	[10]
Apigenin-7-*O*-β-d glucoside	Polyphenol (Flavone glycosides)	flowers, leaves	chloroformic extract	[37]
Apigenin-7-*O*-β-d-glucuronide	Polyphenol (Flavone glycosides)	flowers, leaves	chloroformic extract	[37]
Apigenin-7-*O*-β-d-glucopyranoside	Polyphenol (Flavone glycosides)	flowers	methanolic extract	[36]
Apigenin-7-*O*-β-d-glucuronpyranoside	Polyphenol (Flavone glycosides)	flowers	methanolic extract	[36]
Apigenin-7-*O*-β-d-glucuronpyranoside methyl ester	Polyphenol (Flavone glycosidoester)	flowers	methanolic extract	[36]
Apigenin-7-*O*-β-d-methylglucuronide	Polyphenol (Flavoneglycosidoester)	flowers, leaves	Petrol/chloroform, chloroformic extract	[37,38]
Apigenin-7-*O*-(6′′-*E*-caffeoyl)-β-d-glucoside	Polyphenol (Flavoneglycosidoester)	flowers, leaves	Petrol/chloroform, chloroformic extract	[37,38]
Apigenin	Polyphenol (Flavone)	plants, flowers, leaves	ethanolic extract, methanolic extract, chloroformic extract	[10,31,36,37]
Myricetin	Polyphenol (Flavone)	aerial parts	methanolic extract, DCM extract	[10,22]
Isorhamnetin-3-*O*-β-d-galactoside	Polyphenol (Flavonol glycosides)	flowers, leaves	chloroformic extract	[37,39]
Isorhamnetin-3-*O*-β-d-(6′′-acetyl)-galactoside	Polyphenol (Flavonol glycosides)	flowers, leaves	chloroformic extract	[37,39]
Kaempferol-3-*O*-β-d-glucoside	Polyphenol (Flavonol glycosides)	flowers, leaves	chloroformic extract	[37,39]
Kaempferol 3-β-d-glucopyranoside	Polyphenol (Flavonol glycosides)	aerial parts	methanolic extract, DCM extract	[10]
Isorhamnetin-3-*O*-β-d-glucopyranoside	Polyphenol (Flavonol glycosides)	flowers	methanolic extract	[36]
Isorhamnetin-3-*O*-β-d-glucuronpyranoside	Polyphenol (Flavonol glycosides)	flowers	methanolic extract	[36]
Isorhamnetin-3-*O*-rutinoside	Polyphenol (Flavonol glycosides)	flowers	methanolic extract	[36]
Isorhamnetin-3-*O*-robinobioside	Polyphenol (Flavonol glycosides)	flowers	methanolic extract	[36]
isorhamnetin 3-*O*-β-d-(6′′-acetyl)-galactopyranoside	Polyphenol (Flavonol glycosides)	flowers	ethanolic extract	[17]
Rutin hydrate	Polyphenol (Flavonol)	aerial parts	methanolic extract, DCM extract	[10]
Rutin	Polyphenol (Flavonol)	plants, flowers	ethanolic extract, methanolic extract	[31,36]
Hyperoside	Polyphenol (Flavonol)	plants	ethanolic extract	[31]
Isoquercitrin	Polyphenol (Flavonol)	plants	ethanolic extract	[31]
Guaijaverin	Polyphenol (Flavonol)	plants	ethanolic extract	[31]
Avicularin	Polyphenol (Flavonol)	plants	ethanolic extract	[31]
Quercitrin	Polyphenol (Flavonol)	plants	ethanolic extract	[31]
Quercetin	Polyphenol (Flavonol)	plants, flowers, leaves	ethanolic extract, chloroformic extract	[10,31,37]
Kaempferol	Polyphenol (Flavonol)	plants, flowers, leaves	ethanolic extract, chloroformic extract	[31,37]
Luteolin	Polyphenol (Flavonol)	plants	ethanolic extract	[31]
Genistein	Polyphenol (Isoflavones)	aerial parts	methanolic extract, DCM extract	[10]
Coumaric acid	Polyphenol (Phenylpropanoid)	leaves, flowers	essential oil	[3]
Anethole	Polyphenol (Phenylpropanoid)	leaves, flowers	essential oil	[3]
Eugenol	Polyphenol (Phenylpropanoid)	leaves, flowers	essential oil	[3]
Neochlorogenic acid	Polyphenol (Phenylpropanoid)	plants	ethanolic extract	[31]
Chlorogenic acid	Polyphenol (Phenylpropanoid)	plants	ethanolic extract	[31]
Caffeic acid	Polyphenol (Phenylpropanoid)	plants	ethanolic extract	[10,31]
Vanillic acid	Polyphenol (Phenylpropanoid)	aerial parts	methanolic extract, DCM extract	[10]
*p*-Coumaric acid	Polyphenol (Phenylpropanoid)	aerial parts	methanolic extract, DCM extract	[10]
Coumarin	Polyphenol (Phenylpropanoid)	aerial parts	DCM extract	[10]
Abietatriene	Terpene (Diterpene)	leaves, flowers	essential oil	[3]
α-Pinene	Terpene (Monoterpene)	leaves, flowers, herb	essential oil	[3,6]
β-Pinene	Terpene (Monoterpene)	leaves, flowers, herb	essential oil	[3,6]
β-Myrcene	Terpene (Monoterpene)	leaves, flowers, herb	essential oil	[3,6]
Alloocimene	Terpene (Monoterpene)	leaves, flowers	essential oil	[3]
3-Carene	Terpene (Monoterpene)	leaves, flowers	essential oil	[3]
1,4-Cineole	Terpene (Monoterpene)	leaves, flowers	essential oil	[3]
α-Terpinene	Terpene (Monoterpene)	leaves, flowers	essential oil	[3]
*p*-Cymene	Terpene (Monoterpene)	leaves, flowers	essential oil	[3]
Limonene	Terpene (Monoterpene)	leaves, flowers	essential oil	[3]
β-Phellandrene	Terpene (Monoterpene)	leaves, flowers	essential oil	[3]
1,8-Cineole	Terpene (Monoterpene)	leaves, flowers	essential oil	[3]
*cis*-Ocimene	Terpene (Monoterpene)	leaves, flowers	essential oil	[3]
*trans*-Ocimene	Terpene (Monoterpene)	leaves, flowers	essential oil	[3]
*cis*-Linalooloxide	Terpene (Monoterpene)	leaves, flowers	essential oil	[3]
*trans*-Linalooloxide	Terpene (Monoterpene)	leaves, flowers	essential oil	[3]
α-Terpinolene	Terpene (Monoterpene)	leaves, flowers	essential oil	[3]
Linalool	Terpene (Monoterpene)	leaves, flowers, herb	essential oil	[3,6]
1,3,8-*p*-Menthatriene	Terpene (Monoterpene)	leaves, flowers	essential oil	[3]
*cis*-Pinenehydrate	Terpene (Monoterpene)	leaves, flowers	essential oil	[3]
*trans*-Pinocarveol	Terpene (Monoterpene)	leaves, flowers	essential oil	[3]
*trans*-Pinenehydrate	Terpene (Monoterpene)	leaves, flowers	essential oil	[3]
Camphor	Terpene (Monoterpene)	leaves, flowers	essential oil	[3]
Pinocarvone	Terpene (Monoterpene)	leaves, flowers	essential oil	[3]
Citronellal	Terpene (Monoterpene)	leaves, flowers	essential oil	[3]
Lavandulol	Terpene (Monoterpene)	leaves, flowers	essential oil	[3]
4-Terpineol	Terpene (Monoterpene)	leaves, flowers	essential oil	[3]
α-Terpineol	Terpene (Monoterpene)	leaves, flowers	essential oil	[3]
Dihydrocarveol	Terpene (Monoterpene)	leaves, flowers	essential oil	[3]
*cis*-Piperitol	Terpene (Monoterpene)	leaves, flowers	essential oil	[3]
*trans*-Piperitol	Terpene (Monoterpene)	leaves, flowers	essential oil	[3]
*trans*-Carvenol	Terpene (Monoterpene)	leaves, flowers	essential oil	[3]
Nerol	Terpene (Monoterpene)	leaves, flowers	essential oil	[3]
*cis*-Carveol	Terpene (Monoterpene)	leaves, flowers	essential oil	[3]
Isogeraniol	Terpene (Monoterpene)	leaves, flowers	essential oil	[3]
Pulegone	Terpene (Monoterpene)	leaves, flowers	essential oil	[3]
Neral	Terpene (Monoterpene)	leaves, flowers	essential oil	[3]
Piperitone	Terpene (Monoterpene)	leaves, flowers	essential oil	[3]
*cis*-Sabinenehydrate acetate	Terpene (Monoterpene)	leaves, flowers	essential oil	[3]
Geraniol	Terpene (Monoterpene)	leaves, flowers	essential oil	[3]
Linalyl acetate	Terpene (Monoterpene)	leaves, flowers	essential oil	[3]
*cis*-Verbenyl acetate	Terpene (Monoterpene)	leaves, flowers	essential oil	[3]
Geranial	Terpene (Monoterpene)	leaves, flowers	essential oil	[3]
Geranic acid methyl ester	Terpene (Monoterpene)	leaves, flowers	essential oil	[3]
Thymol	Terpene (Monoterpene)	leaves, flowers	essential oil	[3]
*trans*-Verbenyl acetate	Terpene (Monoterpene)	leaves, flowers	essential oil	[3]
*cis*-Pinocarveyl acetate	Terpene (Monoterpene)	leaves, flowers	essential oil	[3]
*trans*-Pinocarveyl acetate	Terpene (Monoterpene)	leaves, flowers	essential oil	[3]
Sabinyl acetate	Terpene (Monoterpene)	leaves, flowers	essential oil	[3]
Dihydrocarveyl acetate	Terpene (Monoterpene)	leaves, flowers	essential oil	[3]
Lavandulyl acetate	Terpene (Monoterpene)	leaves, flowers	essential oil	[3]
α-Terpinenyl acetate	Terpene (Monoterpene)	leaves, flowers	essential oil	[3]
Neryl acetate	Terpene (Monoterpene)	leaves, flowers	essential oil	[3]
Geranyl acetate	Terpene (Monoterpene)	leaves, flowers, herb	essential oil	[3,6]
Geranylacetone	Terpene (Monoterpene)	leaves, flowers, herb	essential oil	[3,6]
Neryl propionate	Terpene (Monoterpene)	leaves, flowers	essential oil	[3]
propionate	Terpene (Monoterpene)	leaves, flowers	essential oil	[3]
Neryl angelate	Terpene (Monoterpene)	leaves, flowers	essential oil	[3]
Geranyl angelate	Terpene (Monoterpene)	leaves, flowers	essential oil	[3]
Carvacrol	Terpene (Monoterpene)	herb, flowers	essential oil	[6]
(*Z*)-β-Ocimene	Terpene (Monoterpene)	herb, flowers	essential oil	[6]
*trans*-*p*-Menth-2-en-1-ol	Terpene (Monoterpene)	herb, flowers	essential oil	[6]
*cis*-Piperitone oxide	Terpene (Monoterpene)	herb, flowers	essential oil	[6]
Piperitenone oxide	Terpene (Monoterpene)	herb, flowers	essential oil	[6]
Nerylacetate	Terpene (Monoterpene)	herb, flowers	essential oil	[6]
δ-Elemene	Terpene (Sesquiterpene)	leaves, flowers	essential oil	[3]
α-Cubebene	Terpene (Sesquiterpene)	leaves, flowers	essential oil	[3]
Naphthalene-1,2,3,4,4a,7-hexahydro-1,6-Dimethyl-4-(1-methylethyl)	Terpene (Sesquiterpene)	leaves, flowers	essential oil	[3]
α-Ylangene	Terpene (Sesquiterpene)	leaves, flowers	essential oil	[3]
α-Copaene	Terpene (Sesquiterpene)	leaves, flowers, herb	essential oil	[3,6]
β-Patchoulene	Terpene (Sesquiterpene)	leaves, flowers	essential oil	[3]
β-Bourbonene	Terpene (Sesquiterpene)	leaves, flowers	essential oil	[3]
β-Cubebene	Terpene (Sesquiterpene)	leaves, flowers, herb	essential oil	[3,6]
β-Caryophyllene	Terpene (Sesquiterpene)	leaves, flowers, herb	essential oil	[3,6]
Aromadendrene	Terpene (Sesquiterpene)	leaves, flowers	essential oil	[3]
α-Himachalene	Terpene (Sesquiterpene)	leaves, flowers, herb	essential oil	[3,6]
α-Humulene	Terpene (Sesquiterpene)	leaves, flowers, herb	essential oil	[3,6]
*cis*-β-Farnesene	Terpene (Sesquiterpene)	leaves, flowers, herb	essential oil	[3,6]
allo-Aromadendrene	Terpene (Sesquiterpene)	leaves, flowers	essential oil	[3]
γ-Muurolene	Terpene (Sesquiterpene)	leaves, flowers	essential oil	[3]
Curcumene-ar	Terpene (Sesquiterpene)	leaves, flowers	essential oil	[3]
β-Selinene	Terpene (Sesquiterpene)	leaves, flowers	essential oil	[3]
δ-Selinene	Terpene (Sesquiterpene)	leaves, flowers	essential oil	[3]
α-Selinene	Terpene (Sesquiterpene)	leaves, flowers	essential oil	[3]
Germacrene B	Terpene (Sesquiterpene)	leaves, flowers	essential oil	[3]
α-Longipinene	Terpene (Sesquiterpene)	leaves, flowers	essential oil	[3]
α-Muurolene	Terpene (Sesquiterpene)	leaves, flowers	essential oil	[3]
α-Farnesene	Terpene (Sesquiterpene)	leaves, flowers, herb	essential oil	[3,6]
γ-Cadinene	Terpene (Sesquiterpene)	leaves, flowers	essential oil	[3]
2,4,6-Trimethylazulene	Terpene (Sesquiterpene)	leaves, flowers	essential oil	[3]
δ-Cadinene	Terpene (Sesquiterpene)	leaves, flowers, herb	essential oil	[3,6]
4,5,9,10-Dehydroisolongifolene	Terpene (Sesquiterpene)	leaves, flowers	essential oil	[3]
Neophytadiene	Terpene (Sesquiterpene)	leaves, flowers, aerial parts	essential oil, methanolic extract	[3,11]
Torreyol	Terpene (Sesquiterpene)	leaves, flowers	essential oil	[3]
*cis*-Chyrsanthenylacetat	Terpene (Sesquiterpene)	leaves, flowers	essential oil	[3]
γ-Himachalene	Terpene (Sesquiterpene)	herb, flowers	essential oil	[6]
Germacrene-d	Terpene (Sesquiterpene)	herb, flowers	essential oil	[6]
Hexahydrofarnesyl acetone	Terpene (Sesquiterpene)	herb, flowers	essential oil	[6]
Copaborneol	Terpene (Sesquiterpene)	herb, flowers	essential oil	[6]
Cyclosativene	Terpene (Sesquiterpene)	herb, flowers	essential oil	[6]
Eremophylene	Terpene (Sesquiterpene)	herb, flowers	essential oil	[6]
Bicyclogermacrene	Terpene (Sesquiterpene)	herb, flowers	essential oil	[6]
Epi-cubebol	Terpene (Sesquiterpene)	herb, flowers	essential oil	[6]
Cubebol	Terpene (Sesquiterpene)	herb, flowers	essential oil	[6]
Caryophyllene oxide	Terpene (Sesquiterpene)	herb, flowers	essential oil	[6]
1-epi-Cubenol	Terpene (Sesquiterpene)	herb, flowers	essential oil	[6]
Decylbutirate		herb, flowers	essential oil	[6]
Stigmasta-7,22-dien-3-ol		aerial parts	methanolic extract	[11]
2-Phytene		aerial parts	methanolic extract	[11]
Methyl syringate 4-*O*-β-d-glycopyranoside		flowers	methanolic extract	[36]
(*Z*)-3-Hexenyl β-d-glucopyranoside		flowers	methanolic extract	[36]

## 4. Conclusions

Additionally, in the papers mentioned above, three studies dealing with the isolation and structure elucidation of several compounds in daisy flowers were complemented to finalize the collection of described substances (Table 1) [36,37,38,39]. There were 319 compounds found and listed, but because of unclear or not fully indicated chemical names, we propose fewer substances, probably around 310, especially flavonoid glycosides, namely apigenin glycosides, which have to be mentioned here. “Compound 1” in Karakas et al. (2014) was not named by the authors either by IUPAC nomenclature or via trivial name and is consequently not listed in Table 1. The distribution of the substance classes is presented in a sunburst plot (Figure 2), in which the terpenoids are the biggest group of compounds followed by polyphenols and lipids.

The aim of the studies summarized here was heterogeneous. There were studies that only analyzed biological activity without the investigation of its ingredients. On the other side, active extracts were examined according to the compounds contained. But, it remains unclear whether one, several, or which of those are responsible for its activity. In some studies, isolated compounds are individually tested for their biological or chemical activity. The activity-guided fractionation workflow, rarely used so far in *B. perennis* research, represents a promising tool to disclose the corresponding bioactive compounds or their mixtures. This approach considers the initial concentration in the extract/plant/organ as well as the interaction, namely masking/additive and/or synergistic effects. This offers the opportunity for detailed structure–activity studies, deciphering the correlation from a certain bioactivity to its compound(s), and should be further investigated in the future.

As daisy flowers were also used in many dishes, e.g., soups, salads, desserts, and starters, the collected data also highlight the importance in the field of food chemistry. *B. perennis* could be an important part of our daily diet, and this review helps to open our minds to this very common occurrence, but it is a widely distributed and underestimated flower. So far, the extracts of *B. perennis* have been tested in vitro, in ovo, and in vivo for their biological activity. With the exception of two homeopathic studies, no studies investigated the effects in humans. This could be a possibility for further research based on the comprehensive results of this review.

## Supplementary Materials

The following supporting information can be downloaded at: https://www.mdpi.com/article/10.3390/molecules28237716/s1, Table S1 contains information on: compound, Substances, class, extract and the literature as well as organs.
